# In situ magnetic separation of antibody fragments from *Escherichia coli* in complex media

**DOI:** 10.1186/1472-6750-13-44

**Published:** 2013-05-20

**Authors:** Martin Cerff, Alexander Scholz, Matthias Franzreb, Iris L Batalha, Ana Cecilia A Roque, Clemens Posten

**Affiliations:** 1 Institute of Life Science Engineering, Division of Bioprocess Engineering, Karlsruhe Institute of Technology (KIT), Karlsruhe, Germany; 2 Institute of Functional Interfaces, Karlsruhe Institute of Technology (KIT), Karlsruhe, Germany; 3 REQUIMTE, Departamento de Quimica, Faculdade de Ciências e Tecnologia, Universidade Nova de Lisboa, 2829-516 Caparica, Portugal

**Keywords:** In situ product removal, Magnetic separation, Protein purification, Affinity ligands, Triazine beads, Metal chelate beads, Recombinant scFv antibody fragments, Extracellular protein, *Escherichia coli* fermentation, Complex media

## Abstract

**Background:**

In situ magnetic separation (ISMS) has emerged as a powerful tool to overcome process constraints such as product degradation or inhibition of target production. In the present work, an integrated ISMS process was established for the production of his-tagged single chain fragment variable (scFv) D1.3 antibodies (“D1.3”) produced by *E. coli* in complex media. This study investigates the impact of ISMS on the overall product yield as well as its biocompatibility with the bioprocess when metal-chelate and triazine-functionalized magnetic beads were used.

**Results:**

Both particle systems are well suited for separation of D1.3 during cultivation. While the triazine beads did not negatively impact the bioprocess, the application of metal-chelate particles caused leakage of divalent copper ions in the medium. After the ISMS step, elevated copper concentrations above 120 mg/L in the medium negatively influenced D1.3 production. Due to the stable nature of the model protein scFv D1.3 in the biosuspension, the application of ISMS could not increase the overall D1.3 yield as was shown by simulation and experiments.

**Conclusions:**

We could demonstrate that triazine-functionalized beads are a suitable low-cost alternative to selectively adsorb D1.3 fragments, and measured maximum loads of 0.08 g D1.3 per g of beads. Although copper-loaded metal-chelate beads did adsorb his-tagged D1.3 well during cultivation, this particle system must be optimized by minimizing metal leakage from the beads in order to avoid negative inhibitory effects on growth of the microorganisms and target production. Hereby, other types of metal chelate complexes should be tested to demonstrate biocompatibility. Such optimized particle systems can be regarded as ISMS platform technology, especially for the production of antibodies and their fragments with low stability in the medium. The proposed model can be applied to design future ISMS experiments in order to maximize the overall product yield while the amount of particles being used is minimized as well as the number of required ISMS steps.

## Background

Process integration such as in situ product removal (ISPR) has emerged as a valuable tool to increase the overall process yield and aims at minimizing costs. ISPR describes the separation of any target from the bioreaction media, e.g. by adsorption of the target to functionalized surfaces [1] in order to minimize production limitations. These can be proteolytic degradation, inhibition of target functionality and target production [2,3]. Magnetic separation was introduced to selectively adsorb the target product to the surface of functionalized magnetic carrier particles [4]. This technique allows for a high product purity in only one step minimizing overall process costs [5]. Potential targets can be proteins [6,7], DNA [8] or microorganisms [9,10]. In situ magnetic separation (ISMS) can further increase the overall target protein yield by separating the target protein itself [11] or removing unwanted molecules from the biosuspension during the bioprocess [12,13]. Ligands known from column chromatography can be employed for functionalization of the beads [6,14].

In this work the overall impact of integrated ISMS on the production of his-tagged single chain fragment variable lysozyme-specific antibody fragments (scFv) D1.3 (furthermore named “D1.3”) from *E. coli* cultivations is investigated. Two types of particles were tested: metal-chelate and triazine-functionalized magnetic beads. Immobilized metal affinity ligands such as Co^2+^, Zn^2+^, Ni^2+^ or Cu^2+^ that chelate to covalently-bound iminodiacetic acid (IDA) are capable of specifically binding histidine residues of his-tagged target proteins. According to the literature these ligands offer important advantages such as chemical stability, high binding capacity, protein recovery, and the possibility of matrix regeneration [15]. The removal (all further examples refer to non-in situ applications) of monoclonal antibodies from the biosuspension with magnetic metal chelate particles has been successfully evaluated by Morgan et al. [16]. Biomimetic affinity ligands based on the triazine scaffold, as the artificial proteins A and L, could also be successfully immobilized on magnetic supports and provide a cost-efficient alternative to isolate IgG antibodies [17,18]. In the current work the triazine beads were tested for the first time to separate scFv D1.3 fragments, corroborating evidence already obtained from theoretical studies [19]. As shown by Holschuh et al., antibodies were successfully captured from biosuspension with MagPrep® Protein A functionalized magnetic beads after the cultivation process [20]. Lysozyme, the antigen of the D1.3, has also been immobilized on magnetic beads to capture Fv antibody fragments from clarified *E. coli* lysate [21]. Small affinity ligands such as IDA charged with divalent metal ions or triazine functionalization are advantageous over biospecific ligands such as protein A due to lower manufacturing costs [6], milder elution conditions, higher stability with regards to leakage and disinfection[18]. However, the use of divalent metal ions as ligands bears the risk to intoxicate microorganisms, especially when they are applied during cultivation [18,22].

To our knowledge, this study is the first in order to test whether ISMS with metal chelate and triazine beads is compatible with the microbial production system and is suitable to increase the overall D1.3 yield. Evaluation of ISMS was performed by comparison of cultivations with and without ISMS supported by process modeling to gain a deeper understanding of the integrated process.

## Methods

### Microorganism and media

Extracellular his-tagged lysozyme-specific scFv D1.3 antibody fragments [23] were produced by the recombinant *E. coli* pOPE101 strain kindly provided by Prof. Dr. Stefan Dübel (Institute of Biotechnology, Technical University Braunschweig) [24,25]. Production of D1.3 (molecular weight ~ 26–27 kDa) was induced by 50 μmol/L isopropyl β-D-1-thiogalactopyranoside (IPTG) while 100 mg/L Na-ampicillin served as selection marker in all media. Three different media were applied. Medium (1) [g/L]: glucose: 20–30; yeast extract: 10; casamino acids: 5; KH_2_PO_4_: 2.3; K_2_HPO_4_: 12.5. The yeast extract consisted of 65.9% (w/w) amino acids, 0.74% and 1.10% (w/w) cysteine and methionine, respectively. Casamino acids consisted of 100% amino acids, 2.51% (w/w) were methionine. Medium (2) contained additional 1.5 g/L MgSO_4_∙7H_2_O and tracer [in mg/L] according to the literature [26] furthermore referred to as “extra salts”: Na_2_MoO_4_∙4H_2_O: 3.1; CoCl_2_∙6H_2_O: 3.1; MnCl_2_∙4H_2_O: 18.8; CuCl_2_∙2H_2_O: 1.9; H_3_BO_3_: 3.8; Zn-acetate∙2H_2_O: 10. Medium (3) is similar to medium (1) with 16 g/L tryptone instead of casamino acids and was only used in shaking flask cultivations. The reactor feed solution contained 200 g/L glucose, 20 g/L casamino acids, 0.1 g/L Na-Ampicillin and 50 μmol/L IPTG provided in one bottle. Chemicals were purchased from Roth (Germany).

### Cultivation conditions in shaking flasks and bioreactor

5 mL cultures were grown as inoculum in either medium (1), (2) or (3). Inoculation of 195 mL of the corresponding medium followed in 500 mL baffled shaking flasks. Each culture was kept at 37°C and 150 rpm for 12 hours (Multitron incubator by Infors, Switzerland) while the pH decreased from pH 7.3 to 6.3 during cultivation. One shaking flask culture was either used as inoculum of 1.8 L fresh medium in the bioreactor (K4-K11) or further used for small scale ISMS experiments (SK1-SK3). The 3.7 L stirred tank bioreactor (model KLF, Bioengineering, Switzerland) was prepared for fed-batch mode equipped with an EasyFerm pH electrode and the OxyFerm amperometric pO_2_ sensor (both Hamilton, Switzerland). Cultivations were performed at pH 7 (addition of 4 M NaOH and 4 M HCl), and the pO_2_ kept above 20% oxygen saturation at 500 rpm stirrer speed. Aeration was realized by a constant flow of 2.5 L/min into the bioreactor. CO_2_ and O_2_ off-gas analysis was performed by the exhalyzer system (Bioengineering, Switzerland). Liquid offline samples could be taken manually or by using the auto sampler 851-AS (Jasco, Germany). A two-stage fermentation process was established both in the reactor and shaking flasks (Table 1): cells were grown in batch mode for 8.5-13 h at 37°C until the production of D1.3 was induced by addition of IPTG and the temperature lowered to 25°C not to overload the secretory pathways of *E. coli*[27]. After induction, the reactor medium was supplemented by a continuous nutrient feed at 2–4 g/h (composition see Microorganism and media section) until the end of cultivation to avoid carbon and nitrogen limitations.

**Table 1:** Overview of cultivation and separation conditions

**Cultivation**	**K4**	**K5**	**K7**	**K8**	**K9**	**K10**	**K11**	**SK1**	**SK2**	**SK3**
Medium	(2)	(2)	(2)	(2)	(1)	(1)	(1)	(3)	(1)	(3)
*t*_*IPTG,ΔT*_ [h]	11	11	10	10	10	13	12	3.5	8.5	8.5
*t*_*ISMS*_ [h]	-	-	75/119	78	-	-	54	8.5	12.3	12.3
Ligand	-	-	IDA-2	IDA-2	-	-	IDA-1	IDA-1	triazine 22/8	
*m*_*mp*_ [g]	-	-	28	28	-	-	8	0.2	0.4	0.4

K4 was conducted using the same cultivation parameters and medium as in K5 (results not shown).

Shaking flask cultivations SK1 and SK 3 were performed in medium (3) containing tryptone instead of casamino acids, but 0.2 g IDA-1 beads were applied in SK1 while triazine beads were used in SK3.

### Particle treatment and in situ magnetic separation (ISMS) procedures

**Figure 1:**
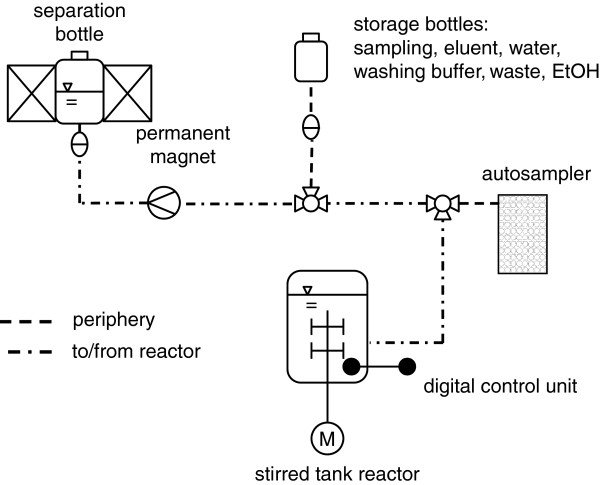
Schematic view of the integrated stirred tank reactor-separator system for the in situ removal of extracellular proteins; D1.3-loaded functionalized magnetic beads were separated from the biosuspension in a 2 L bottle without internal wire matrix when the bottle was placed in the homogenous magnetic field in the gap between the poles of a U-shaped permanent magnet.

Two batches of polyvinyl alcohol (PVA)-coated iminodiacetate (IDA)-functionalized beads were used to chelate Cu^2+^-ions and specifically bind His-tagged D1.3: PVA-IDA-1 (provided by Prof. Franzreb) [28] and PVA-IDA-2 (purchased from Chemagen, Germany). Cu^2+^-ions were chosen as ligands because in the case of IDA-chelating ligands, the ranking of affinities of different metal ions is Cu^2+^ > Ni^2+^ > Zn^2+^ ≥ Co^2+^. These divalent ions are preferably used for purification of histidine-tagged proteins [29]. Triazine-functionalized beads (ligand 22/8) were manufactured according to the literature [17]. Disinfection of the beads and the ISMS system (Figure 1) was done by incubation in 20-30% (v/v) ethanol for 1 hour. IDA-1 and IDA-2 beads were further incubated in 0.05-0.1 mol/L Cu^2+^ solution for 60 min. Elution of the PVA-IDA and triazine beads was performed by applying 0.1 mol/L Na_2_-EDTA/PBS buffer at pH 7.4 or 0.1 mol/L glycine buffer at pH 12.3 for 1 hour, respectively. Before and after each step, at least 3-4 subsequent washing steps of the beads occurred in PBS buffer at pH 7.4 (8.0 g NaCl, 0.2 g KCl, 1.44 g Na_2_HPO_4_∙2 H_2_O, 0.24 g KH_2_PO_4_). Particle treatment was performed at 22°C and 150 rpm in a 2 L bottle (reactor experiments; Multitron incubator by Infors, Switzerland) and in 50 mL Falcon tubes at 20 rpm (shaking flask experiments; bench scale nutating shaker by VWR, USA). Maximum particle concentrations were 70 g/L for disinfection and washing steps as well as 19 g/L and 56 g/L for adsorption and elution steps, respectively. Adsorption was conducted in shaking flasks (50 mL biosuspension) for 10 min, and magnetic separation performed for another 10 min with a NdFeB block permanent magnet (*B*_*r*_ = 1.3 T on the surface; Webcraft, Switzerland) according to the procedure described by Käppler [11]. Adsorption in the closed system (Figure 1) was conducted in a 2 L bottle with 1.5 L biosuspension for at least 30 min. Solid-solid–liquid separation followed for at least 15 min by means of a U-shaped permanent magnet (Steinert, Germany; *B*_*max*_ = 0.45 T in the gap between the pole shoes). In general, adsorption was conducted at 25°C and 150 rpm. After separation, the biosuspension was further cultivated as before. Particles applied in K7 were regenerated and reused in K8. All other beads were only used once (see Table 1).

### Magnetic particle characterization

Equivalent diameters *x*_*50*_ of IDA-1 and IDA-2 particle agglomerates were measured in PBS buffer at pH 7.4 using the Helos analyzer (Sympatec, Germany). The hydrodynamic diameter of triazine particle agglomerates was measured in double distilled (dd)H_2_O using the Zetasizer Nano ZS (Malvern, USA) [17]. Electron microscopy allowed for detailed examination of the primary particles’ structures (FS-SEM 4500/l, Hitachi, Japan; pictures taken at the Institute for Mechanical Process Engineering and Mechanics at KIT). Magnetization properties of the beads were measured with the Micromag 2900 magnetometer (PMC, USA). Batch adsorption studies were carried out with both particle types to determine the binding characteristics of D1.3. Adsorption on triazine and IDA beads was conducted for 20 min and 10-60 min at 22°C on a rotation shaker VV3 (VWR, USA), respectively. Measurements were approximated by the Langmuir adsorption model (Equation 2-1) using a nonlinear least square fitting function (Matlab software, Mathworks, USA) to estimate the maximum adsorption load *Q*_max_^*^ [g of D1.3 per g of beads] and the dissociation constant *k*_*d*_ [g/L]. The equilibrium concentration of D1.3 in the medium after adsorption is *c*_P_^*^ [g/L].

(2-1) $$Q^{*}\;=\;\frac{Q_{\max}^{*}\cdot \;c_{P}^{*}}{c_{P}^{*}\;+\;k_{d}}$$

### Offline analytical procedures

Optical densities of the biosuspension were measured at 600 nm (UV/VIS spectrophotometer UV-2, Unicam, England) and correlated to a BDM standard curve. All buffers were prepared as aqueous solutions. Glucose was determined according to [30] using the GOD-PAP test kit Glucose liquicolor (Human, Germany). Measurements of total protein are based on the method by Lowry modified by Peterson [31] (chemicals from Sigma, USA), and bovine serum albumin was used as calibration standard (albumin fraction V, 8076.2, Roth, Germany). Cu^2+^ concentrations in culture supernatants were measured by inductively-coupled plasma optical emission spectrometry (ICP-OES) [32] using the model JY38S, 1200 W (Horiba Jobin Yvon, France) equipped with a U5000AT + Ultrasonic Nebulizer (Cetac, Omaha, Nebraska 68144 USA). D1.3 was determined by means of an antigen-binding, indirect ELISA-method [25] using 96-well microtiter-plates (Maxisorp, Nunc, Germany). The D1.3 calibration standard was received from a shaking flask culture after adsorption and elution (Ni^2+^-loaded IDA-1 particles, 0.1 mol/L Na_2_-EDTA in PBS, pH 7.4), and was stored at -20°C. According to SDS-PAGE analysis (Coomassie staining; data not shown) the elution fraction was considered to contain scFv D1.3 only, and the D1.3 concentration of the elution sample was then determined with the Lowry method. Elisa plates were coated overnight with 0.5 μg antigen (lysozyme) per well at 4°C. Soluble antibody fragments were detected with anti-c-myc 9E10 antibodies (dilution 1:2000 in MPBST, kindly provided by Dr. Torsten Meyer, Institute of Biotechnology, Technical University of Braunschweig) and anti-mouse IgG antibody conjugated with peroxidase (A2304, Sigma-Aldrich, USA; dilution 1:10000). After treatment with TMB (BioRad, USA) and sulfuric acid, absorbance was measured at 450 nm using a microtiter plate reader (NanoQuant Infinite M200 Pro, Tecan, Germany), and the blank of a sample without D1.3 (at 450 nm) was subtracted. ELISA measurements were performed in the linear range of D1.3 concentration. The rate of D1.3 degradation was examined by incubation of diluted culture supernatant at 25 and 37°C on a rotation shaker and taking samples hourly.

### Modeling the integrated bioprocess on the reactor and cellular level

Mathematical models of the stirred tank reactor and the cells were established in Matlab-Simulink (Mathworks, USA) based on differential equations (time *t* as independent variable), mass balances, kinetics and stoichiometries. Three substrates *S,i* with *i =* glucose (glu), total amino acids (aa) and cysteine (cys) were modeled. The model was established for two reasons. Although many assumptions were made, mainly to describe growth and product formation, it shall provide a deeper understanding of the whole bioprocess. However, the main goal was to provide a tool that can be used to optimize the ISMS steps. This is important because the amount of particles used and the number of required ISMS steps must be minimized because they are connected to extra costs. A set of simulation parameters is proposed in Table 2 and explanation of their origin is provided in the text. Calculation of the reactor volume *V* and the dilution rate *D* considered constant substrate and base flow *q*_*S,i*_ and *q*_*base*_ (*≈ q*_*S,i*_) as well as volume reduction by the auto sampler *q*_*sampler*_ (Equation 3-1 - 3-2). The flow rate was chosen in that way that glucose was not limiting in the reactor.

(3-1) $$\frac{\mathrm{dV}}{\mathrm{dt}}\;=\;\sum_{i}q_{S,i}\;+\;q_{\mathrm{base}}-\;q_{\mathrm{sampler}}$$

(3-2) $$D\;=\;\frac{\sum_{i}q_{S,i}+\;q_{\mathrm{base}}}{V}$$

**Table 2:** Simulation parameter sets for cultivations in medium (1) and (2) without and with additional salts

	***k***_***tr ***_**[m/h]**	***k***_***d ***_**[g/L]**	***Q***_***max ***_**[g/g]**	***A***_***spec ***_**[m**^**2**^**/g]**	***r***_***P,i,0 ***_**[mg g**^**-1**^ **h**^**-1**^**]**	***r***_***P,j,0 ***_**[g g**^**-1**^ **h**^**-1**^**]**	***r***_***P,i,deg ***_**[h**^**-1**^**]**	***r***_***cys ***_**[mg g**^**-1**^ **h**^**-1**^**]**
K5/K7/K8	2.5e-06 ^a1)^	0.039 ^a2)^	0.081 ^a2)^	44 ^*b)*^	0; 1-6 ^c)^	0.003 (*t* > 0) ^c)^	0 ^d)^	0.2 after ~40 h ^c)^
K9/K10/K11	5.95e-05 ^a1)^	0.014 ^a2)^	0.1 ^a2)^	44 ^*b)*^	0; 1 ^c)^	0.003 (*t* > 0) ^c)^	0 ^d)^	0.0 ^c)^
All cultivations	***μ***_***max ***_**[1/h]**	***r***_***m ***_**[g g**^**-1**^ **h**^**-1**^**]**	***m***_***f ***_**[−]**	***q***_***feed ***_**[g/h]**	***q***_***sampler ***_**[mL/h]**	***K***_***M,glu ***_**[g/L]**	***K***_***M,aa (cys) ***_**[g/L]**	***Y***_***X,glu ***_**[g/g]**
	0.37-0.44 ^a3)^	0.2 ^c)^	0.5-0.7 ^b)^	2-4 ^e)^	12.5 ^e)^	0.1 ^b)^	0.01 ^b)^	0.25 ^d)^
	***Y***_***X,aa (cys) ***_**[g/g]**	***Y***_***CO2,X ***_**[g/g]**	***Y***_***CO2,P,i (j) ***_**[g/g]**	***e***_***C,glu ***_**[−]**	***e***_***C,CO2 ***_**[−]**	***e***_***C,X ***_**[−]**	***e***_***C,P,i *(j)**_**[−]**	***e***_***aa,X ***_**[−]**
	1.5 (15) ^c)^	0.45 ^b)^	0.5 ^b)^	0.4	0.27	0.5 ^b)^	0.43 ^b)^	0.64 ^b)^
	***e***_***aa,P,i(j) ***_**[−]**	***e***_***cys,yeast ***_**[−]**	***e***_***cys,X ***_**[−]**	***e***_***cys,P,i ***_**[−]**	***e***_***cys,P,j ***_**[−]**	***E***_***a ***_**[J/mol]**	***A***_***Arrh.***_**[1/h]**	
	1.0 ^c)^	0.0073 ^b)^	0.012 ^b)^	0.028 ^b)^	0 ^c)^	56000 ^b)^	1.2e9 ^c)^	

^a)^ estimated by minimizing least squares for Eqs. ^a1)^ 3-7, ^a2)^ 2-1, ^a3)^ values valid for 37°C; for 25°C calculation according to Equation 3-10.

^b)^ estimated from literature.

^c)^ estimated manually.

^d)^ according to measurements.

^e)^ set manually.

Calculations of the BDM concentration *c*_*X*_ (Equation 3-3) took the specific growth rate *μ* of the microorganisms into consideration which was obtained from Equation 3-9.

(3-3) $$\frac{dc_{x}}{\mathrm{dt}}=\mu \cdot c_{x}-D\cdot c_{x}$$

Substrate concentrations *c*_*S,i*_ were derived from Equation 3-4 where *r*_*S,i*_ are the specific substrate uptake rates which were calculated from Equation 3-8. The composition of the substrate feed (*c*_*s,i,f*_) can be taken from Microorganism and media section. When *i* = cysteine is considered as substrate, the assumption was made that cysteine could be synthesized by the cells at a fixed rate *r*_*cys*_ when additional sulfur (i.e. (NH_4_)_2_SO_4_) was present in the medium (as in K4, K5, K7, K8) and cysteine provided in the medium was exhausted after approx. 40 h of cultivation. Otherwise no cysteine was synthesized (K9, K10, K11).

(3-4) $$\frac{dc_{S,i}}{\mathrm{dt}}=-r_{S,i}\cdot c_{X}-D\cdot c_{S,i}+D\cdot c_{S,i,f}+r_{\mathrm{cys}}\cdot c_{X}$$

Carbon dioxide production (Equation 3-5) depends on the specific carbon dioxide production rate *r*_*CO2*_ of the microorganisms. Hereby, the CO_2_ flux into the reactor was neglected as it is insignificant in comparison to the CO2 produced.

(3-5) $$\frac{dm_{co_{2}}}{\mathrm{dt}}=q_{co_{2}}=r_{co_{2}}\cdot c_{X}\cdot V$$

Concentrations of D1.3 and other proteins *c*_*P,i*_ and *c*_*P,j*_ were calculated from Equation 3-6. While *r*_*P,i,j*_ describe protein formation rates (Equation 3-11) that were estimated manually by approximation of the measurements, *r*_*P,i,deg*_ represents D1.3 degradation which was determined experimentally. Adsorption and removal of proteins from the bioreactor is considered by the term $\dot{\Gamma }\left[g_{i}{}_{j}m^{-}{}^{2}h^{-1}\right]$ that takes particle properties such as the BET-surface *A*_*spec*_ [m^2^ g^-1^] into account [28]. The total protein content was calculated as *c*_*P*,*tot*_ = *c*_*P*,*i*_ + *c*_*P*,*j*_

(3-6) $$\frac{dc_{P,i,j}}{\mathrm{dt}}=r_{P,i,j}\cdot c_{X}-D\cdot c_{P,i,j}-r_{P,i,\deg}\cdot c_{P,i}-\frac{\dot{\Gamma }\cdot m_{\mathrm{mp}}\cdot A_{\mathrm{spec}}}{V}$$

Adsorption kinetics of proteins to a solid matrix is determined by the transport rate *k*_*tr*_ of the protein to the surface and the adsorption rate *k*_*a*_[33]. Equation 3-7 is obtained for the assumption *k*_*a*_ ≫ *k*_*tr*_[m/h] with the surface-specific load of protein on the particle *Γ*[*g*/*m*^2^]. Hereby, *k*_*tr*_ was estimated by minimizing least squares for Equation 3-7. *k*_*d*_ and Q^*^_max_ were estimated from Equation 2-1.

(3-7) $$\frac{\mathrm{d\Gamma}}{\mathrm{dt}}=\dot{\Gamma }=1000\cdot k_{\mathrm{tr}}\cdot \left[c_{P,i}-\frac{k_{d}\cdot \Gamma }{\frac{Q_{\max}^{*}}{A_{\mathrm{spec}}}-\Gamma }\right]$$

The substrate uptake was considered as the first step in the metabolic network and was modeled by Michaelis-Menten kinetics (Equation 3-8). *K*_*M*_ was taken from the literature with the assumption *K*_*M,aa,cys*_ ≈ *K*_*M,NH4+*_[11].

(3-8) $$r_{S,i}=r_{S,i,\max}\cdot \frac{c_{S,i}}{c_{S,i}+K_{M}}$$

Maximum substrate uptake rates were calculated by Equation 3-9 where *Y*_*X,S,i*_ is the integral yield coefficient for BDM on each substrate *i* that was obtained from measurements (for *i* = glucose) or estimated manually (for *i* = total amino acids and cysteine).

(3-9) $$r_{S,i,\left(\max\right)}=\frac{\mu_{\left(\max\right)}}{Y_{X,S,i}}$$

*μ*_*max*_ was experimentally determined for *T* = 37°C and estimated by Equation 3-10 for *T* = 25°C [34]. Hereby, *A*_*Arrh.*_ is a pre-exponential factor that was estimated manually, *E*_*A*_ the activation energy [34], and *R* = 8.314 J mol^-1^ K^-1^ the universal gas constant [35].

(3-10) $$\mu_{\max}=A_{\mathrm{Arrh}.}\cdot \exp\left(\frac{-E_{A}}{R\cdot \left(T+237.15K\right)}\right)$$

D1.3 production was modeled by a constant specific production rate *r*_*P,i,0*_ > 0 after induction in medium (1) (as in K9, K10, K11) and after an additional lag phase (30 h) in medium (2) (as in K5, K7, K8: it was *r*_*P,i,0*_ = 1 mg g^-1^ h^-1^ (K8), 3 mg g^-1^ h^-1^ (K5) and 6 mg g^-1^ h^-1^ (K7)). Production of proteins beside D1.3 was constantly modeled as *r*_*P,j,0*_ > 0 (Equation 3-11).

(3-11) $$r_{P,i,j}=r_{P,i,j,o}$$

The total mass balance for glucose in the anabolism (ana) and catabolism (cata) is modeled as Equation 3-12.

(3-12) $$r_{\mathrm{glu}}=r_{\mathrm{ana}}+r_{\mathrm{cata}}$$

Component balances of carbon (C), amino acids and cysteine in the anabolism are shown in Equations 3-13 - 3-15. Hereby, *e* is the molar or mass fraction of a chemical compound within another compound as indicated. The following values were taken from the literature: *e*_*C,X*_[36], *e*_*C,P,i,j*_[37], *e*_*aa,X*_[38], *e*_*cys,yeast*_ (user manual; Difco yeast extract by voigtglobal, Germany), *e*_*cys,X*_[38]*, e*_*cys,P,i*_ ≈ 6**∙**121.2 g/mol/26000 g/mol [37]. To obtain *e*_*aa,P,i,j*_ the assumption was made that proteins only consist of amino acids while *e*_*cys,P,j*_ was estimated manually.

(3-13) $$e_{C,\mathrm{glu}}\cdot r_{\mathrm{ana}}=e_{C,X}\cdot \mu +e_{C,P,i}\cdot r_{P,i}+e_{C,P,j}\cdot r_{P,j}$$

(3-14) $$r_{\mathrm{aa}}=e_{\mathrm{aa},X}\cdot \mu +e_{\mathrm{aa},P,i}\cdot r_{P,i}+e_{\mathrm{aa},P,j}\cdot r_{P,j}$$

(3-15) $$r_{\mathrm{cys}}=e_{\mathrm{cys},X}\cdot \mu +e_{\mathrm{cys},P,i}\cdot r_{P,i}+e_{\mathrm{cyc},P,j}\cdot r_{p,j}$$

The carbon balance for the respiration is presented in Equation 3-16.

(3-16) $$e_{C,CO_{2}}\cdot r_{CO_{2}}=e_{C,\mathrm{glu}}\cdot r_{\mathrm{cata}}$$

The energy balance couples anabolism and catabolism of the cell, but intracellular ATP levels were not quantified. Hence, the carbon dioxide production rate *r*_*CO2*_ was modeled as a function of specific growth as well as protein production rates (Equation 3-17). Yield coefficients on ATP as well as the maintenance rate *r*_*m*_ were taken from the literature [39,40] and normalized (Equation 3-18). The maintenance rate *m*_*f*_ · *r*_*m*_ (*m*_*f*_ = 1 at *T* = 37°C, *m*_*f*_ < 1 at T < 37°C) refers to intracellular energy requirements. Estimates for *m*_*f*_ were received from [41].

(3-17) $$\begin{array}{l} r_{CO_{2}}=\frac{y_{CO_{2},\mathrm{ATP}}}{y_{X,\mathrm{ATP}}}\cdot \mu +\frac{y_{CO_{2},\mathrm{ATP}}}{y_{P,i,\mathrm{ATP}}}\cdot r_{P,i}\\ \;+\frac{y_{CO_{2},\mathrm{ATP}}}{y_{P,j,\mathrm{ATP}}}\cdot r_{P,j}+m_{f}\cdot r_{m} \end{array}$$

(3-18) $$\begin{array}{l} Y_{CO_{2},X}=\frac{Y_{CO_{2},\mathrm{ATP}}}{y_{X,\mathrm{ATP}}};\\ Y_{CO_{2},P,i}=\frac{Y_{CO_{2},\mathrm{ATP}}}{Y_{P,i,\mathrm{ATP}}};Y_{CO_{2},P,j}=\frac{Y_{CO_{2},\mathrm{ATP}}}{Y_{P,j,\mathrm{ATP}}} \end{array}$$

Mathematic modeling of D1.3 production requires solutions for nine rates *r* by means of 11 available equations (Equations 3-8 - 3-18). Thus, the system would be over-determined if all equations were used [42], and rates might possibly contradict each other. However, there can only be one specific growth rate that relies upon the limiting substrate and its uptake rate. Thus, the three theoretical substrate uptake rates *r*_*glu*_, *r*_*aa*_, *r*_*cys*_ were determined separately before the system matrix was calculated. If glucose is assumed to be limiting, the remaining eight rates were calculated by finding a solution for the equation system A∙s = b with the stoichiometric matrix A and the vectors s = [*r*_*ana*_*, r*_*cata*_*, r*_*aa*_*, r*_*cys*_*, μ, r*_*P,i*_*, r*_*P,j*_, *r*_*CO2*_] and b. Finally, the set of rates corresponding to the minimum solution of *μ* was chosen for further calculation (*r* and *c* > 0). Two different parameter sets for K5-K8 (with extra salts) and K9-K11 (without extra salts) were applied (Table 2). The main difference is found in the parameters *r*_*P,i,0*_ that were obtained due to different D1.3 production rates and *r*_*cys*_ due to different medium compositions.

## Results and discussion

### Magnetic particle characterization

Equivalent diameters of particle agglomerates *x*_*50*_ = 1.0 [28] and 2.2 μm were found for IDA-1 and IDA-2 beads in PBS buffer at pH 7.4. The hydrodynamic diameter of triazine bead agglomerates was 0.33 ± 0.03 μm [17]. Results obtained from electron microscopy also revealed much smaller primary particles (11 ± 1 nm for triazine beads [17] and 100–400 nm for IDA 2). Thus, these particles have a very high surface-to-volume ratio which is desirable to obtain a high protein binding capacity [6]. It is known from the literature that magnetic particles with a diameter ≥ 0.5-1 μm can be easily separated by simple magnetic separators (e.g. a glass bottle without wire matrix and a permanent magnet as used in this work), while separation of smaller particles is much more efficient when high gradient magnetic separators are used (with internal wire matrix) [6,43]. This applies because the magnetic force which is experienced by the particles through the external magnetic field is proportional to the particle volume and the gradient of the magnetic field strength inside the separation chamber [6]. Especially at higher viscosities of the cultivation medium (above 1 mPa∙s) magnetic particle agglomerates are favorable due to shorter separation times [44]. Since particle agglomeration can occur spontaneously in the medium, especially at high particle concentrations [6], it is important for in situ protein separation that no biomass, i.e. microorganisms, is included or adsorbed in/to those agglomerates. Losses of biomass, i.e. *Escherichia coli* cells, after the ISMS step are discussed in Comparison of the total D1.3 yield in processes with and without ISMS section.

Saturation magnetization *M*_*S*_ = 40 A m^2^ kg^-1^[28] and 24.6 ± 1.5 A m^2^ kg^-1^ were measured for IDA-1 and IDA-2 beads, and *M*_*S*_ = 37.7 ± 2.2 A m^2^ kg^-1^ for triazine beads. The remanent magnetization was low at *M*_*R*_ = 0.13-1.3 A m^2^ kg^-1^, thus particles were considered superparamagnetic. It is important to keep in mind that magnetic particles or agglomerates thereof have superparamagnetic properties only when the size of each individual magnetite core is between 15–50 nm [14,45].

**Figure 2:**
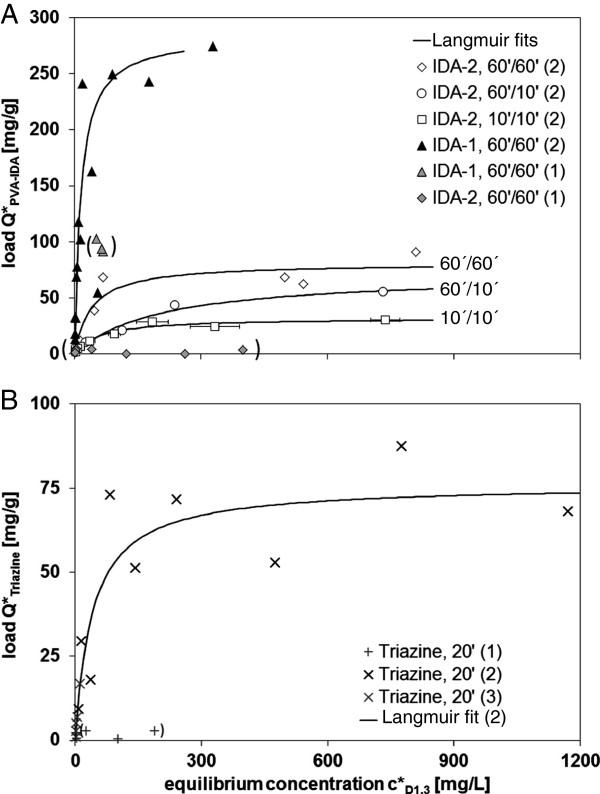
**Isotherms for scFv D1.3 on (A) PVA-IDA-1 and −2 and (B) triazine-functionalized beads; explanations: 60’/60’ means 60 min incubation of the IDA-beads in 0.1 M Cu**^**2+**^**-solution and 60 min for adsorption in the biosuspension; while media (1) and (3) do not contain extra salts (see Microorganism and media section), extra salts were added to medium (2); data in brackets was not fitted by the Langmuir model.**

Adsorption of the target protein scFv D1.3 to the beads was quantified by measuring isotherms (Figure 2A and B) that could be approximated by the Langmuir adsorption model (Equation 2-1). In the experiments maximum loads of *Q*_max_^*^ = 0.28 g/g and 0.08 g/g were found for IDA-1 and IDA-2 beads, respectively, when medium (2) containing extra salts was used (Figure 2A). In medium (1) without extra salts, *Q*_max_^*^ was significantly decreased to ~ 0.004-0.1 g/g (Figure 2A). Different maximum loads might be based on different particle production processes and are not further discussed. Highest loads of IDA beads were achieved for 60 min incubation in Cu^2+^-solution and 60 min adsorption. For triazine beads we found *Q*_max_^*^ = 0.08 g/g in medium (2) with extra salts (Figure 2B). According to the low *k*_*d*_-values obtained from the isotherms (*k*_*d*_ = 0.01-0.04 g/L), all beads can be categorized as affinity adsorbents [6]. The maximum obtained load for IDA-1 beads *Q*_max_^*^ = 0.28 g/g is very high compared to *Q*_max_^*^ = 0.13 g/g for green fluorescent protein [28]. Possibly, D1.3 concentrations were overestimated by the ELISA procedure in general (see Offline analytical procedures section, preparation of the calibration standard) due to incomplete elution or loss of D1.3 activity in the elution buffer (for discussion see Comparison of the total D1.3 yield in processes with and without ISMS section.). However, the proposed ELISA method is sufficient to compare the results provided in this study among each other.

### Degradation of the scFv D1.3 in cultivation media

The stability of the D1.3 was tested in cell-free cultivation medium up to 50 h of incubation, but no degradation or any loss of D1.3 activity could be detected (results not shown). Although the D1.3 fragments were found to be stable in cell-free medium, ISMS was applied to study its impact on the whole bioprocess in presence of the microorganisms with the particle systems characterized in Magnetic particle characterization section.

### Production of D1.3 and ISMS in bioreactor cultivations

#### Process performance in complex medium with extra salts

**Figure 3:**
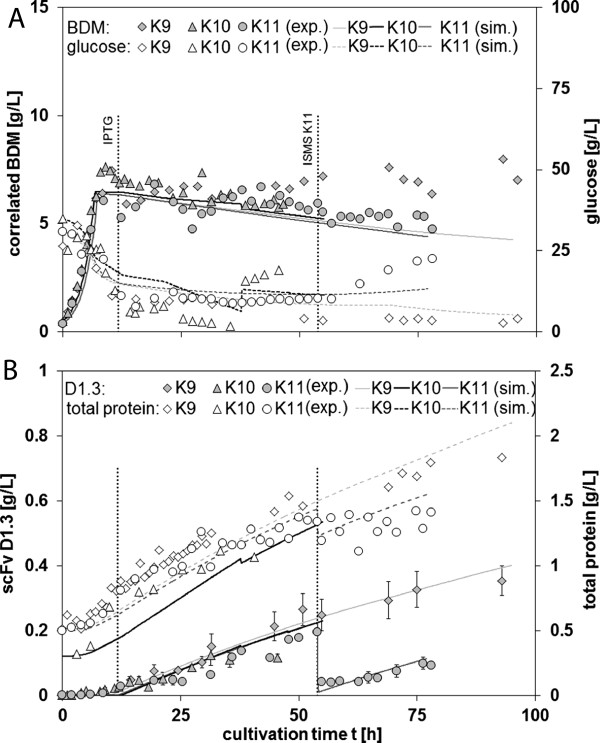
**ISMS of D1.3 by means of PVA-IDA-2 magnetic particles from medium (2) containing extra salts; (A) correlated BDM and glucose as well as (B) normalized D1.3 and total protein concentrations over cultivation time (measurements = exp.); D1.3 was normalized to the concentration *c***_***D1.3***_**before the first ISMS step at ~ 75 h for better comparison [g/L]: 0.59 (K5), 0.22 (K8), 1.87 (K7); vertical dashed lines: induction by IPTG and ISMS steps; all other lines: simulated data (sim.); total protein was also simulated but not shown for better visibility.**

Two reference cultivations K4 and K5 were conducted without and two cultivations K7 and K8 carried out with ISMS to investigate the performance of (repeated) ISMS (Table 1). Results of K4 were similar to those in K5 and are not shown. Before induction, cells were grown in medium (1) up to a bio dry mass concentration of 5–7.5 g/L at a maximum specific growth rate of *μ*_*max*_ = 0.35-0.44 h^-1^ while glucose was rapidly consumed but not limiting (Figure 3A). After induction, BDM concentrations increased up to 11.5-12.8 g/L at the end of K5, K7 and K8. Offline BDM and glucose concentrations were similar in cultivations with and without ISMS (Figure 3A).

D1.3 was only produced after induction: in K5, K7 and K8, D1.3 production was delayed by 30 hours (Figure 3B). Further investigations are required to clarify whether the delay was due to inhibition of D1.3 production by the additional salts provided in the medium. Although cultivations K5, K7 and K8 were performed in the same medium under the same conditions, maximum obtained D1.3 concentrations differed significantly before the ISMS step (0.22 g/L, 0.59 g/L and 1.87 g/L in K8, K5 and K7, respectively). According to Harrison, different leakage rates of the D1.3 fragments from the periplasmic space into the medium might be responsible for those differences [46]. Furthermore, the obtained extracellular D1.3 titers are much higher compared to those reported in literature (*c*_*D1.3*_ = 0.04 g/L) [45]. Although D1.3 concentrations might have been overestimated within the ELISA procedure, the impact of ISMS on the process could be studied well. For better comparison of processes with and without ISMS, D1.3 concentrations were normalized to the final D1.3 concentration before the ISMS step.

In K7 and K8 92% and 98% of the overall D1.3 were separated from the medium by ISMS, respectively (Figures 3B). However, no significant production was observed after separation in K7 and K8. Instead, D1.3 activities decreased towards zero until the end of cultivations. This effect cannot be attributed to significant degradation of the D1.3 fragments in the medium since no D1.3 degradation could be quantified. Thus, D1.3 production was supposed to be inhibited as a consequence of the ISMS step. Further explanation is provided in the Section Response of the microorganisms to the ISMS steps. During all cultivations, protein other than D1.3 was continuously produced and secreted into the medium up to 2 g/L.

#### Process performance in complex medium without extra salts

**Figure 4:**
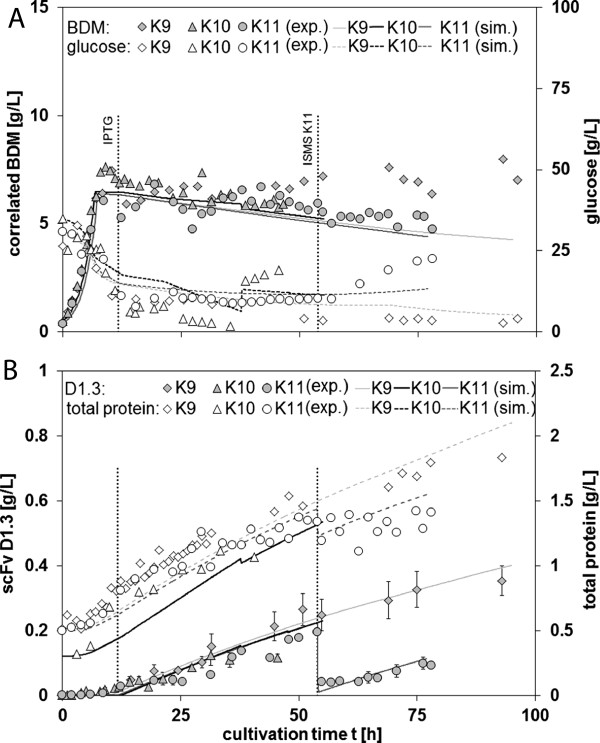
ISMS of D1.3 by means of PVA-IDA-1 magnetic particles from medium (1) containing no extra salts; (A) correlated BDM and glucose as well as (B) D1.3 and total protein concentrations over cultivation time (measurements = exp.); vertical dashed lines: induction by IPTG and ISMS steps; all other lines: simulated data (sim.).

Two reference cultivations K9 and K10 were conducted without ISMS (Table 1). BDM and glucose concentrations (Figure 4A) as well as total protein concentrations are comparable to those obtained from cultivations in medium containing extra salts (Figures 3A and B). However, after induction, BDM concentrations remained constant in K9 or slightly decreased in K10-K11. D1.3 was immediately produced after ISMS in K11 (Figure 4B), and maximum D1.3 concentrations before the ISMS step were lower compared to the results obtained from K5-K8 (0.2 g/L in K11), and they did not increase above 0.12-0.35 g/L at the end of cultivations (K9-K11). Investigations of the culture medium suggest that the lower overall BDM and D1.3 concentrations are based on potential sulfur limitation in medium (1) after induction (K9-K11). Only methionine was supplied by the feed being theoretically sufficient to deliver the sulfur required for D1.3 production. Sulfur is a compound of cysteine that is required for biomass and protein synthesis. Calculations were based on 0.2% (w/w) sulfur and 2.8% (w/w) cysteine content of the BDM [36] and D1.3 fragments [37], respectively. In K11 78% of the overall D1.3 were separated from the medium by ISMS (Figure 4B). In contrast to K7 and K8, D1.3 was produced in K11 after ISMS reaching 50% of the maximum level before separation.

### Validation of the process simulation by offline data

**Figure 5:**
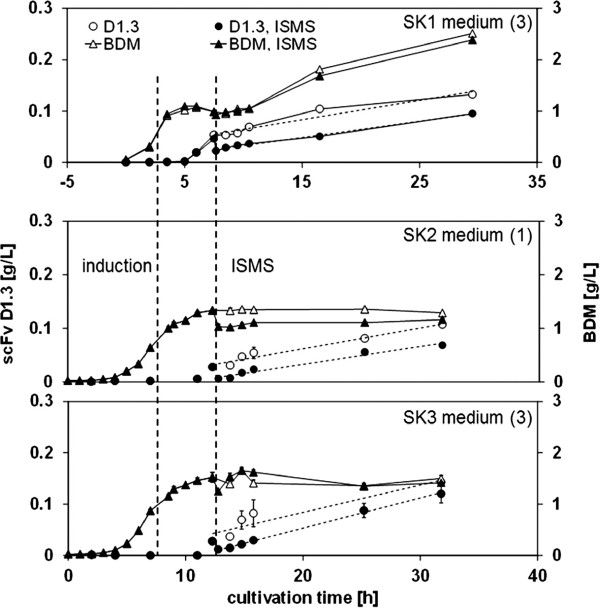
ISMS of D1.3 by means of IDA-1 beads (upper panel) and triazine beads (middle and lower panels) in different media from shaking flask cultivations.

Simulation of BDM and glucose concentrations over cultivation time could be validated by offline measurements for all cultivations (Figures 3A, 4A). As it was observed in all cultivations, biomass growth already declined before induction. The experimentally unknown limitation was modeled as cysteine, i.e. sulfur limitation (data not shown), and exceptionally as glucose limitation in K7 after 98 hours. Cells were considered capable of producing cysteine from sulfate that was abundant in the medium of K5, K7 and K8 to fulfill the requirements of D1.3 production and biomass growth (*r*_*cys*_ > 0 after *t* ≈ 40 h), but that was not abundant in K9, K10 and K11 (*r*_*cys*_ = 0 after induction). This can be an explanation for decreased BDM and D1.3 titers in cultivations K9-K11. Degradation of D1.3 in the medium was set to zero (*r*_*P,i,deg*_ = 0, see Degradation of the scFv D1.3 in cultivation media section). Significant differences between the D1.3 offline data and the simulation occurred after separation in K7 and K8. While D1.3 was reproduced in the simulation, no D1.3 reproduction occurred in the offline data (Figure 3B). Simulated D1.3 and total protein courses could be validated well with experimental data in K9-K11 including the ISMS step. D1.3 production rates were comparable before and after ISMS (K11) but were not elevated compared to the reference cultivations K9 and K10 (Figure 4B). Similar results were obtained for cultivations with and without ISMS in shaking flask cultivation (SK1, Figure 5 IDA-1 particles were applied in medium (3)). In the best case similar D1.3 production rates before and after ISMS were expected because no degradation of the D1.3 was observed. In the Response of the microorganisms to the ISMS steps an explanation is given why the production of D1.3 might have been limited in K7 and K8 after the ISMS steps.

### Response of the microorganisms to the ISMS steps

**Figure 6:**
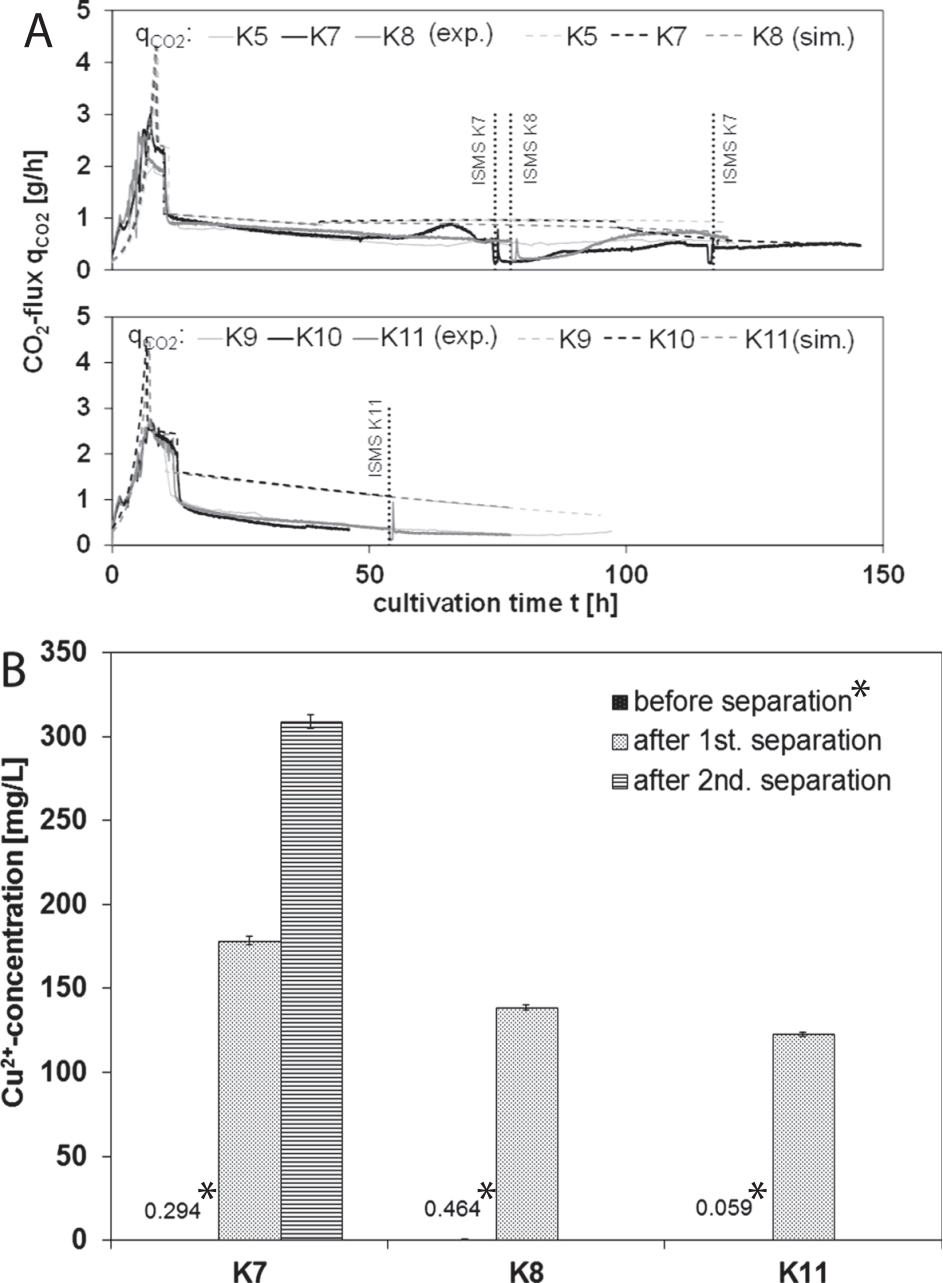
**(A) Comparison of CO**_**2 **_**off-gas fluxes from the bioreactor in medium (2) (upper panel) and medium (1) (lower panel); solid lines: measured data (exp.); dashed lines: simulated data (sim.); (B) determination of Cu**^**2+ **^**in the medium before and after ISMS; asterisks: Cu**^**2+**^**-concentrations in the medium before ISMS (medium control).**

Measurements of CO_2_ in the off-gas and Cu^2+^ concentrations in the medium were conducted to study the culture viability before and after ISMS. Decreased or no D1.3 production after ISMS correlates well with a decreased respiratory capacity of the cells: in K7 and K8, the CO_2_ fluxes in the off-gas stream dropped from 0.60 to 0.15 g/h and from 0.56 to 0.17 g/h after the initial ISMS step indicating a decreased metabolic activity. In K7, *q*_*CO2*_ recovered up to 0.34 g/h after 87 h of cultivation (Figure 6A). Interestingly, after 87 h of cultivation, BDM concentrations almost doubled although no significant D1.3 production was observed any more. This implies that cells were either inhibited to produce D1.3 after ISMS or the process suffered from microbial contamination. The second ISMS step in K7 did not evoke a drop of the CO_2_ signal at 116 h. In K11, no significant drop of the CO_2_ signal was observed indicating that the metabolic activity was not decreased by ISMS (Figure 6A). Overestimation of the CO_2_ flux by the simulation (Figure 6A) might be based on the fact that no overflow metabolism (e.g. acetate formation) was taken into account in the model [47].

Measurements of Cu^2+^ concentrations in the medium revealed significant copper contamination of up to 180 mg/L and 300 mg/L after the first and second ISMS step in K7, respectively (Figure 6B). Copper accumulation in the medium due to insufficient washing steps after copper-loading of IDA particles seems unlikely because four subsequent washing steps were carried out. Partial leakage of copper from the beads most likely occurred during their application in the medium. This suggestion is supported by high availability of up to 0.4 mmol free IDA groups on the particle surface per gram PVA-IDA-2 beads that theoretically chelate equimolar amounts of Cu^2+^ ions (see specification of the beads). These findings imply that cell metabolism and thus production of D1.3 by recombinant *E. coli* can be strongly inhibited by free Cu^2+^ ions. Cu^2+^ ions might be reduced in the fermentation medium to Cu^+^ ions that are able to structurally damage proteins and decrease their activity due to highly reactive radical intermediates [29]. Copper concentrations below 120 mg/L as in K11 seem to be less inhibiting, and D1.3 production was less diminished in K11 compared to K7 and K8 where copper concentrations exceeded 140 mg/L. In SK1, the copper concentration was not measured before and after ISMS. However, due to similar concentrations of Cu^2+^-loaded IDA-1 particles in the biosuspension during the ISMS step (5.33 g/L in K11 and 4 g/L in SK1), it can be assumed that the copper concentration in the biosuspension after ISMS in SK1 did not exceed the value obtained in K11 (*c*_*Cu*_ = 120 mg/L). In literature, the minimal inhibitory concentration of Cu^2+^, Ni^2+^, Co^2+^ and Zn^2+^ ions on *E. coli* cells is 1.0 mmol/L (*c*_*Cu*_ = 63.6 mg/L) [22]. Further research can clarify if a potential D1.3 production inhibition can be circumvented by using other divalent metal ions: Ni^2+^ and Co^2+^ ions are reported to be less carcinogenic than Cu^2+^ ions [48] but are still potentially harmful if intended for therapeutic purposes [29]. Since Zn^2+^ is redox-inactive and less toxic than Cu^2+^, it might be tested as metal ligand to bind his-tagged D1.3 [22]. Another approach would be to use tetradendate chelating agents such as nitrilotriacetic acid (NTA) instead of tridendate ligands such as IDA to obtain more stable complexes with the metal ion [29] and potentially avoid excessive leakage of Me^2+^ into the medium.

### Cultivation and ISMS in shaking flasks by means of triazine beads

Additional ISMS experiments were conducted in shaking flasks using triazine-functionalized beads. In both complex media (1) and (3) (Figure 5 SK2 and SK3, respectively), up to 57-79% of D1.3 was separated from the medium, and D1.3 was produced after ISMS. For both media, similar linearized D1.3 production rates *r*_*P,i,0*_ = 0.003-0.004 g g^-1^ h^-1^ were found. These results are comparable to those obtained for IDA-1 beads used in SK1 (Figure 5). However, no copper or other heavy metal ions were required for adsorption using the triazine beads. We have thus shown experimentally that scFv D1.3 antibody fragments can be selectively separated from biosuspension by gum arabic coated magnetic carrier particles functionalized with the triazine ligand 22/8, a biomimetic of protein A. Until now, binding of those beads has only been reported to full IgG antibodies [17] although it has been shown recently that ligand 22/8 can also bind to the Fab portion of IgG by molecular modeling studies [19].

### Comparison of the total D1.3 yield in processes with and without ISMS

**Figure 7:**
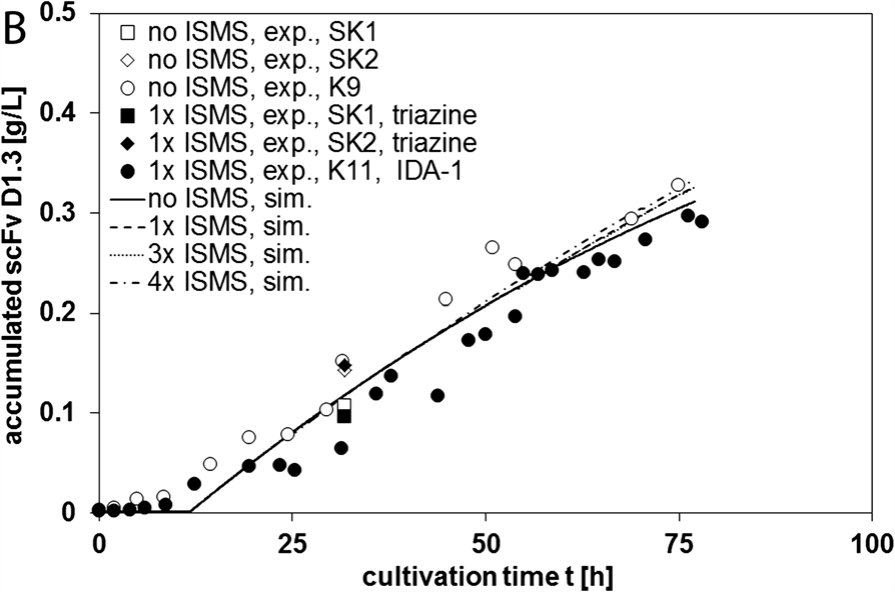
Overall comparison of the accumulated D1.3 concentrations obtained from cultivations with and without ISMS; symbols: measurements; lines: simulation (parameters from K11); theoretical simulated ISMS steps were equally distributed between 12 and 78 h.

As can be seen from Figure 7, ISMS did not yield extra benefit for total D1.3 production since the total accumulated D1.3 concentrations with and without ISMS were equal in the best case. This result has been confirmed by simulation including theoretical multi step ISMS. As shown for extracellular protease that was degraded in biosuspension [49] such a model can be useful to optimize the number of ISMS steps before the experiment is actually performed. Neither an inhibition of D1.3 in the medium on D1.3 production itself nor losses of D1.3 degradation were observed in the extracellular medium. While the total D1.3 yield without ISMS was set *Y*_*no ISMS*_ = 1, the yield with ISMS was *Y*_*ISMS*_ = 0.91-1.1 and 0.64-0.84 for IDA and triazine beads, respectively. Elution efficiencies between 7 and 100% were received for both PVA-IDA-1 and -2 particle charges. It remains unclear whether low elution efficiencies are to be attributed to incomplete elution or loss of activity in the elution buffer although EDTA is reported to be a strong eluent which extracts the metal ions from IDA and disrupts interactions between proteins and chelating ligands [29]. Adsorption and elution efficiency of his-tagged proteins from IDA-functionalized magnetic beads can depend on the chelated metal ion: in literature, multi-subunit adsorption of a his-tagged protein onto chelated Cu^2+^ was reported which significantly limited elution efficiency [50]. Furthermore, the isolated target protein can potentially be structurally damaged in presence of reduced Cu^+^ ions which leads to reduced activity [29,50]. This implies to test other divalent metal ions such as Ni^2+^, Zn^2+^ or Co^2+^ which might lead to higher elution efficiencies [50]. Elution efficiencies of D1.3 from triazine-functionalized beads only reached 6%. In comparison, elution efficiencies of 35% were obtained for IgG [17], and the elution protocol needs further improvement. SDS-PAGE should be applied to control the purity of the elution samples.

Losses of biomass due to the adsorption procedure were 9 ± 2% and 21 ± 4% for IDA and triazine beads, respectively. This indicates that biomass is more attracted to adsorb to the triazine beads. Coating the beads with an extra layer of biopolymer such as alginate might minimize the risk of bio mass adsorption [14]. The application of a high gradient magnetic separator can further help to minimize biomass losses because formation of one single compact filter cake with inclusion of biomass is avoided.

## Conclusions

Results have shown that PVA-IDA particles are well suited to separate and purify his-tagged proteins from complex media (maximum purification factors of 3.2-11.7). However, their application for ISMS is limited due to the release of chelated copper ions into the medium which have shown to inhibit D1.3 production above *c*_*Cu*_ > 120 mg/L. Other less toxic divalent metal ions should be tested and validated as ligands for the IDA-beads. But it must be shown in semi-continuous ISMS experiments whether this particle type proofs to be compatible with the bioprocess or is better suited for separation of his-tagged proteins after cultivation as it is well known from the literature. In the case of separation of antibodies and their fragments, triazine-functionalized magnetic particles were found to be an excellent low-cost alternative to protein A-functionalized beads. Although no negative impact of these beads was observed on the bioprocess (the loss of triazine ligands into the medium remains unknown), further semi-continuous ISMS experiments are necessary. In the best case, similar D1.3 production rates were observed before and after in situ separation when IDA- or triazine-functionalized particle types were applied. No degradation of D1.3 was measured in biosuspension and, thus, ISMS did not increase the total yield of the D1.3-production process as was shown by experiments and simulation. Under consideration of the obtained results and proposed improvements on PVA-IDA particle performance, both PVA-IDA and triazine-functionalized magnetic beads can serve as ISMS platform technology to obtain highly purified product in only one separation step. In situ magnetic separation can bring most benefit to those production processes where antibodies or their fragments lack stability in the medium or are subject to degradation. These processes need to be identified. This study provides a route how to proceed with experiments and optimize the process by simulation: small scale experiments should be conducted first and followed by scale up. When the kinetics of degradation or lack of stability of the target are quantified and implemented into the model, the latter can be applied to optimize ISMS in order to maximize the overall product yield while the amount of particles being used is minimized as well as the number of required ISMS steps.

## Abbreviations

Greek symbols

*μ*: Specific growth rate, g g^-1^ h^-1^; *Γ*: Surface-specific particle load, g m^-2^;

Latin symbols

A*_Arrh_*: Arrhenius pre-factor; A*_Spec_*: BET-surface, m^2^ g^-1^; *B*: Magnetic flux density, T; *c*: Concentration, g L^-1^; *D*: Dilution rate, h^-1^; *e*: Mass or molar fraction; *E_A_*: Activation energy, J mol^-1^; *k_a_*: Adsorption rate, m h^-1^; *k_d_*: Dissociation constant, g L^-1^; *K_M_*: Michaelis-Menten constant, g L^-1^; *k_tr_*: Transport rate, m h^-1^; *M*: Saturation magnetization, A m^2^ kg^-1^; *m*: Mass, g; *m_f_*: Maintenance factor; *Q*: Adsorption load, g g^-1^; *q*: Flow to/from reactor, L/h, g/h; *r*: Specific uptake/production/maintenance rate, g g^-1^ h^-1^; *t*: Cultivation time, h; *T*: Temperature,°C; V: Reactor volume, L; *x_50_*: Equivalent diameter, m; *Y_X,S,i_*: Integral yield coefficient, g g^-1^; Y: Overall product yield; *y_X,S,i_*: Differential yield coefficient, g g^-1^.;

Indices

*: Equilibrium; *0*: Initial condition; *aa*: Amino acids; *ana*: Anabolism; *ATP*: Adenosine triphosphate; *C*: Carbon; *cata*: Catabolism; *CO_2_*: Carbon dioxide; *Cu^2+^*: Divalent copper ions; *cys*: Cysteine; *deg*: Degradation; *f*: Feed; *glu*: Glucose; ISMS: In situ magnetic separation; *m*: Maintenance; *max*: Maximum; *mp*: Magnetic particles; *P,i*: scFv D1.3; *P,j*: Other proteins besides D1.3; *R*: Remanent; S: Saturation; *S,i*: Substrate; *X*: Biomass.;

Natural constant

 R: Universal gas constant, 8.314 g mol^-1^ K.;

Other abbreviations

BDM: Bio dry mass; BET: Brunauer-Emmett-Teller; ISMS: In situ magnetic separation; IPTG: Isopropyl β-D-1-thiogalactopyranoside; scFV: Single chain fragment variable; PBS: Phosphate buffered saline; MPBST: Modified phosphate buffered saline with Tween 20; PVA-IDA: Polyvinyl alcohol-iminodiacetate.

## Competing interest

The authors declare that they have no competing interest.

## Authors’ contributions

MC conducted the cultivations and magnetic separation experiments, implemented the reactor-cell model and drafted the manuscript. AS contributed to the experiments and provided valuable discussion. MF provided the MPVA-IDA-1 functionalized magnetic particles and assisted with the HGMS setup. ACAR supervised the preparation of the triazine particles and provided valuable discussion. ILB carried out the synthesis of the triazine-functionalized magnetic particles. ACAR and ILB helped to design the adsorption and elution experiments. CP conceived and initiated the project, and assisted with the model. All authors carried out the revision of the manuscript. All authors read and approved the final manuscript.
